# Electrophysiological characteristics of neuropathic pain model in mice and a technique to evaluate peripheral nerve damage in the sciatic nerve

**DOI:** 10.1016/j.heliyon.2025.e42879

**Published:** 2025-02-20

**Authors:** Hidenori Suzuki, Masahiro Funaba, Sayaka Ogi, Kosei Matsumoto, Norihiro Nishida, Kazuhiro Fujimoto, Takashi Sakai, Kenji Nishikawa

**Affiliations:** a Department of Orthopaedics Surgery, Yamaguchi University Graduate School of Medicine, Yamaguchi, Japan; b Pharmaceutical Research Laboratory, Pharmaceutical Division, UBE Corporation, Ube, Japan

**Keywords:** Neuropathic pain, Sensory nerve action potentials (SNAPs), Compound motor action potentials (CMAPs), C57BLKS/J lar-+Leprdb/+Leprdb (db/db) mice, Paclitaxel-induced experimental neuropathic pain models

## Abstract

**Background:**

Experimental neuropathic pain (NP) models were developed from peripheral injuries and are widely used for pain research. However, previously published methods and outcomes in the evaluation of sciatic nerve dysfunction vary widely across each report. In this study, we established the electrophysiological analysis of sensory nerve action potentials (SNAPs) and compound motor action potentials (CMAPs) as objective methods for evaluating sciatic nerve dysfunction and revealed the electrophysiological characteristics of NP mice models.

**Methods:**

We used male C57BL/6 J mice, C57BLKS/J lar- + Leprdb/+Leprdb (db/db) mice, and mouse models of paclitaxel-induced NP and spared nerve injury (SNI) surgery (10 mice in each group). For nerve conduction studies, we used evoked potential/electromyography apparatuses and bipolar electrodes as stimulating and recording electrodes for SNAPs and CMAPs. SNAPs were recorded from the sciatic nerve. For the distal motor latency and CMAPs amplitude measurements, we placed the cathode of the recording electrode on the gastrocnemius muscle.

**Results:**

In electrophysiological assessments, the SNI model showed the most severe sensory disturbance in SNAP with allodynia in the sciatic nerve. The db/db model showed moderate sensory disturbance with allodynia, whereas the paclitaxel model showed slight sensory disturbance but also with allodynia. Only the SNI model showed significantly lower amplitudes of CMAPs than those of normal mice, whereas the paclitaxel and db/db models showed sensory disturbance but no loss of motor function.

**Conclusions:**

We focused on the detailed methodology of electrophysiological assessment in experimental NP models. Our method of electrophysiological analysis of SNAPs and CMAPs of the sciatic nerve was well reflected in the pain withdrawal tests performed in each NP model and may be useful for future research of NP in experimental NP models.

## 1 Introduction

Previously published articles in recent years have reported on several kinds of experimental models of peripheral nerve damage and neuropathy in the sciatic nerve for analysing pain mechanisms, treating neuropathic pain (NP), axonal regeneration, and remyelination [[1], [2], [3], [4], [5], [6]]. Experimental models of NP were developed as a result of peripheral trauma or metabolic or toxic injuries. These experimental models promote sensory disorders that use peripheral neuropathy to induce pain [[7], [8], [9], [10], [11]]. However, as NP is not directly measurable in animals, researchers infer the intensity of pain by observation of ‘pain-like’ behaviours such as withdrawing from nociceptive stimuli [6,12].

Electrophysiological analyses of sensory nerve action potentials (SNAPs) and compound motor action potentials (CMAPs) are used as the most objective methods for the evaluation of peripheral nerve dysfunction [4,[13], [14], [15], [16]]. However, previously published methods and outcomes in the evaluation of sciatic nerve dysfunction have varied widely across each report [[2], [3], [4], [5],^10^,^11^,[13], [14], [15], [16], [17], [18], [19], [20], [21], [22], [23], [24], [25], [26], [27], [28], [29]]. Therefore, it is difficult to accurately evaluate sciatic nerve function electrophysiologically and objectively following nerve dysfunction and functional recovery when we create and treat experimental models of NP.

In this study, we established a method of electrophysiological analysis of SNAPs, sensory nerve conduction velocity (SCV), and CMAPs for universal and easy analysis of sciatic nerve function for future research of regenerative therapy for NP. In addition, we revealed the electrophysiological characteristics of NP mice models, C57BLKS/J lar- + Leprdb/+Leprdb (db/db) mice, a paclitaxel-induced neuropathic pain (PI-NP) model, and a spared nerve injury (SNI) surgery model, which are the most frequently used major experimental NP models.

We used three different mouse models of NP to show the characteristics of peripheral nerve dysfunction in nerve conduction studies (NCS). The analysed models included C57BL/6 J mice as a model of normal function and C57BLKS/J lar- + Leprdb/+Leprdb (db/db) mice for analysis of peripheral nerve damage in a diabetes model, PI-NP model, and SNI surgery model that respectively represented a model of normal sciatic nerve function and those of diabetic neuropathy (DN), chemotherapy-induced peripheral neuropathy (CIPN), and entrapment neuropathy (EN) [[3], [4], [5], [6], [7],^10^,^11^,[14], [15], [16], [17], [18], [19], [20],^22^,^23^,^25^,^28^]. DN, CIPN, and EN are the main targets of medication in clinical settings; however, recent treatments used for DN, CIPN, and EN are not adequate for resolving chronic pain in patients with NP. We as clinicians are eager to develop a radical treatment of patients with these NPs. To develop several new therapies, we have to verify the effectiveness of new treatments using experimental NP models before clinical trials. Therefore, we need to establish a methodology of evaluation of experimental NP models. Previous electrophysiological evaluations of experimental NP models differed widely among species, methodology, and outcomes. Therefore, in this study, we focused on the methodology in electrophysiological assessment and devised a stable and quantitative evaluation method.

In the present study, we showed our established methods of electrophysiological analysis of CMAPs, SNAPs, and SCV in the sciatic nerve according to clinical electrophysiological methods [[30], [31], [32]]. We also revealed the electrophysiological characteristics and the outcome of pain withdrawal tests of experimental NP mice models of DN, CIPN, and EN.

We advanced the electrophysiological evaluation of experimental NP models by incorporating SNAPs alongside CMAPs, thereby enabling an independent and detailed assessment of sensory nerve function. This multimodal approach addresses a significant limitation in prior studies, in which the methodologies often lacked reproducibility and focused predominantly on motor function, leaving sensory dysfunction underexplored [[3], [4], [5], [6], [7],^10^,^11^,[14], [15], [16], [17], [18], [19], [20],^22^,^23^,^25^,^28^]. By integrating SNAPs into the evaluation framework, we established a robust methodology that quantifies the extent of sensory nerve damage with precision, offering valuable insights into its relationship with pain behaviours such as allodynia and hyperalgesia.

Furthermore, we optimized the technical protocols by standardizing stimulation and recording techniques, minimizing artefacts, and ensuring reliable, reproducible data across different experimental conditions. This rigorous framework not only enhances the reliability of electrophysiological assessments but also provides a universal foundation for evaluating peripheral nerve dysfunction in diverse NP models. By addressing the gaps in the existing methodologies, our work can pave the way for more accurate preclinical evaluation of novel therapies for sensory and motor neuropathies, ultimately bridging the translational gap toward clinical applications.

## 2 Methods

The Animal Care and Use Committee of Pharmaceuticals Research Laboratory, UBE Corporation (Ube, Japan), approved all animal care and experimental procedures (approval numbers: 22135, 22141–22143). All methods were reported in accordance with ARRIVE guidelines (https://arriveguidelines.org), and all methods were carried out in accordance with relevant guidelines and regulations. For animal welfare, we performed this study with the minimum required sample size in each group from a statistical perspective. In addition, we conducted all of the experiments with the utmost consideration to avoid causing pain and stress in the experimental animals.

### 2.1 Animals

Male C57BL/6 J mice (n = 10, aged 10–11 weeks and weighing 23–26 g) and C57BLKS/J lar- + Leprdb/+Leprdb (db/db) mice (n = 10, aged 11 weeks and weighing 36–45 g) were purchased from Japan SLC, Inc. (Shizuoka, Japan). They were housed in pathogen-free conditions at a constant temperature of 23 ± 2 °C and humidity of 50 % ± 10 %, under a 12-h light/dark cycle, and had access to water and standard pellet food ad libitum. Animals were acclimated for at least five days before use.

All efforts were made to minimize suffering. All SNI mice received an analgesic (carprofen, 5 mg/kg, subcutaneously) immediately after the operation and the next day after surgery. Each mouse was monitored until awake and moving freely around the recovery chamber. Animals were then housed singly for the duration of the study. The method of euthanasia consisted of administration of an overdose of sodium pentobarbital (>100 mg/kg) injected intraperitoneally.

### 2.2 Spared nerve injury surgery

SNI surgery was performed in C57BL/6 J mice (n = 10, aged 7 weeks) under general anaesthesia of 2–3% isoflurane in oxygen as previously described [8]. After incising the skin on the left lateral surface of the thigh, we divided the biceps femoris muscle and spread it lengthwise to allow exposure of the three sciatic nerve branches. After identifying the common peroneal and tibial nerves and ligating them with 8-0 silk, we transected them distal to the ligation and removed a 2–4-mm length of each nerve. We took care to ensure that the sural nerve remained intact and to avoid any contact with or stretching of this nerve. We then closed the muscle and skin layers and returned the animals to their cages and littermates. We performed electrophysiological testing for analysis of the SNI mice (n = 10, aged 11 weeks and weighing 22–26 g) 4 weeks after the SNI surgery.

### 2.3 Model of paclitaxel-induced neuropathic pain

Paclitaxel (Tokyo Chemical Industries Co., Ltd., Tokyo, Japan) was dissolved in Cremophor ELP and ethanol to 11 mg/mL in a 1:1 (v/v) mixture and then diluted with 0.9 % NaCl to 1.1 mg/mL immediately before dosing. The paclitaxel solution (5.5 mg/kg) was injected intraperitoneally in C57BL/6 J mice (n = 10, aged 6 weeks) every other day on days 0, 2, and 4. We performed electrophysiological testing to analyse the paclitaxel mice (n = 10, aged 10 weeks and weighing 22–24 g) 4 weeks after the first paclitaxel injection.

### 2.4 Nerve conduction studies

For NCS, we used evoked potential/electromyography apparatuses (Neuropack S1, MEB-9404MB; NIHON KOHDEN, Tokyo, Japan) along with bipolar electrodes (hooked type, UM2-5050-3 and silver pointed type, UL2-2020-3; UNIQUE MEDICAL, Tokyo, Japan) as stimulating and recording electrodes for SNAPs and CMAPs. During the NCS, room temperature was above 25 °C, and skin temperature was measured at the thigh and kept above 36 °C. Following induction, anaesthesia was maintained at 2–3% with a flow rate of 1 L/min O_2_.

**Fig. 1:**
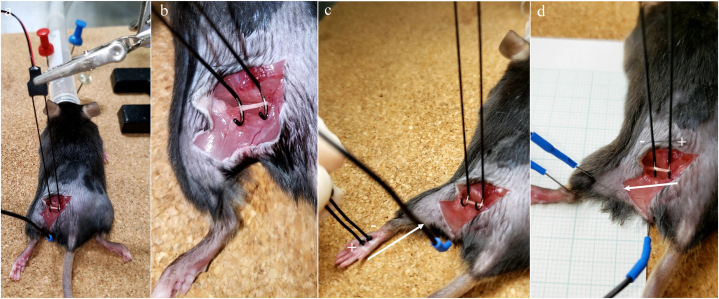
Photographs of mouse preparation. **a.** Electrophysiological assessment in whole mouse position and the positioning of electrodes on the mouse sciatic nerve. **b.** We used hooked-type bipolar electrodes for stimulating and recording of sensory nerve action potentials and compound motor action potentials. **c.** Photographs showing NCS. For NCS, we used evoked potential/electromyography apparatuses. In addition, we used hooked-type and silver pointed-type bipolar electrodes for stimulating and recording of SNAPs. **d.** CMAPs were recorded at the gastrocnemius. We placed the cathode of the recording electrode on the skin overlying the centre portion of the gastrocnemius muscle of the hind limb and the anode at the ankle joint. For stimulation of the sciatic nerve at the proximal hind limb, two hooked pointed-type stimulators were used as the cathode and anode. **c, d.** The reference needle electrode was inserted in the skin over the base of the tail. CMAPs, compound motor action potentials; SNAPs, sensory nerve action potentials; NCS, nerve conduction studies.

The mice were anaesthetized with inhaled isoflurane and placed in the prone position. We performed through-hip dissection under a magnifying lens and exposed the sciatic nerve between the sciatic notch and its entrance point into the muscles. The invasive surgical technique included only skin incision and exposure of the sciatic nerve without muscle damage and bleeding because the sciatic nerve was visible just under skin. This technique caused little pain in the mice and resulted in a less invasive examination. We then mounted the nerve in situ in a Teflon hemi-tube equipped with two stimulating, two recording, and one earth platinum electrode (Fig. 1a and b, hooked type), paying special attention to place the stimulating and recording electrodes after the platinum electrode. SNAPs were recorded from the sciatic nerve using the hook-type electrode after orthodromic stimulation of the plantar nerve using the silver point-type stimulator (Fig. 1c, silver pointed type). We used an S5 Grass stimulator to deliver rectangular voltage pulses (0.5-ms duration at 0.2-Hz frequency), and we increased the stimulus intensity by 100 % after the maximal response was obtained at the recording site. To avoid causing stress and pain to the experimental animals, six doctors specializing in animal surgery collaborated and performed this experiment in 10 min.

The extracellular SNAP signals were fed through a high-gain, low-noise amplifier and stored in an oscilloscope for determination of their latencies and amplitudes (Fig. 1c). Peak-to-peak SNAPs amplitudes were measured and averaged at least 20 times. Latency was measured from stimulus onset to negative onset of the SNAP. SCV was calculated using the classical method of dividing distance by latency. NCS were performed three times on each mouse, and the average value was calculated.

### 2.5 CMAPs recorded at gastrocnemius

Following SNAPs recording, we placed the cathode of the recording electrode on the skin above the central part of the hind limb gastrocnemius muscle, with the anode at the ankle joint, and inserted the reference needle electrode on the skin at the base of the tail. We used two hook pointed-type stimulators as the cathode and anode to stimulate the sciatic nerve at the proximal hind limb (Fig. 1d). To avoid directly injuring the sciatic nerve or other structures, we took care not to insert the stimulating electrodes too close to the sciatic nerve or too deeply. CMAPs responses were acquired by increasing stimulus intensity until the response amplitude stopped increasing. Then, to ensure supramaximal stimulation and to obtain a maximal and additional response, we increase the stimulation to ∼120 % of the stimulus intensity used. We recorded this response as the maximal CMAP if it did not further increase in size. We measured distal motor latency from stimulus onset to the onset of the CMAP and CMAPs amplitude from baseline to its peak, following which we calculated the average values of 3 CMAPs tests. We measured distal latency from the stimulus artefact to the initial CMAP onset and calculated nerve conduction velocity (NCV) as the distance measured with an inextensible tape along the course of the sciatic-tibial peripheral nerve between the distal and proximal cathode stimulation sites divided by the difference between the two latencies.

After the SNAPs and CMAPs recordings were completed and anaesthesia was discontinued, we continued to monitor the mice until they regained sufficient consciousness to maintain sternal recumbency. Following the NCS, the mice were euthanized by isoflurane overdose until respiration ceased.

### 2.6 Assessment of pain behaviour

We also performed plantar withdrawal and von Frey withdrawal tests to confirm the NP of each model in terms of thermal hyperalgesia and mechanical allodynia. We evaluated the thermal pain threshold (Hargreaves test) by measuring the latency of paw withdrawal after applying a thermal stimulus with a model 390G thermal stimulator (IITC Life Sciences, Woodland Hills, CA, USA). Mice were placed in a plexiglass cage positioned over a glass plate at a temperature of 32 °C and allowed to acclimate for at least 1 h. The thermal stimulator was then placed under the glass plate, and the projection bulb was focussed directly at the middle plantar surface of the left hind paw. We measured paw withdrawal latency (PWL) three times at 5–10-min intervals and calculated the mean latency for each mouse. To prevent tissue damage, we set a cut-off time of 20 s. For the von Frey withdrawal test, mice were placed into a plexiglass cage on a wire-mesh floor and acclimated for at least 1 h. We recorded paw withdrawal threshold (PWT) with an electronic von Frey model 2390 Aesthesiometer (IITC Life Sciences). The rigid electronic von Frey hair was applied to the midplantar surface of the left hind paw, and the maximum force at the point that elicited a withdrawal response was recorded as the PWT. As with PWL, we measured PWT three times at 5–10-min intervals and calculated the mean latency for each mouse.

### 2.7 Statistical analyses

The data of the SNAPs, CMAPs, SCV, PWL, and PWT parameters were analysed with one-way ANOVA. Multiple comparisons were performed with Dunnett's multiple comparison, as appropriate, following the Kruskal-Wallis test using StatFlex Ver. 7 for Windows (Artec, Osaka, Japan; https://www.statflex.net/). A *P*-value of <0.05 was considered to indicate statistical significance.

## 3 Results

### 3.1 Electrophysiological characteristics of NP mice models in nerve conduction studies

#### 3.1.1 Sensory nerve action potentials

**Fig. 2:**
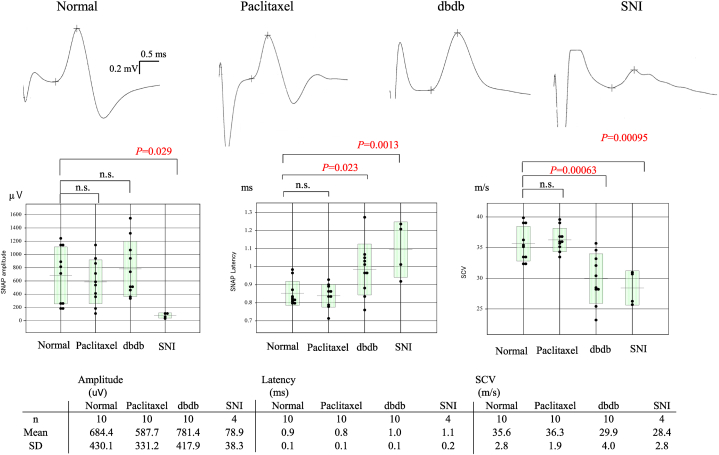
SNAPs in the sciatic nerve. Latency and amplitude of SNAPs in normal and NP mice models of CIPN (paclitaxel), DN (db/db), and EN (SNI) are shown. Mice with EN (SNI) had significantly lower SNAPs amplitude (P = 0.029), longer SNAPs latency (*P* = 0.0013), and lower SCV (*P* = 0.00095) than normal mice. Mice with DN (db/db) had significantly longer SNAPs latency (*P* = 0.023) and lower SCV (*P* = 0.00063) than normal mice. SNAPs amplitude, SNAPs latency, and SCV in the CIPN (paclitaxel) model were not significantly different from those of the normal mice. The data of the SNAPs and SCV parameters were analysed with one-way ANOVA. Multiple comparisons were performed with Dunnett's multiple comparison, as appropriate, following the Kruskal-Wallis test. CIPN, chemotherapy-induced peripheral neuropathy; DN, diabetic neuropathy; EN, entrapment neuropathy; NP, neuropathic pain; n.s., not significant; SCV, sensory conduction velocity; SNAPs, sensory nerve action potentials; SNI, spared nerve injury.

We measured SNAPs in the sciatic nerve (Fig. 1a–c). The latency and amplitude of SNAPs in the normal and NP mice models of CIPN (paclitaxel), DN (db/db), and EN (SNI) are shown in Fig. 2.

The EN (SNI) model showed significantly lower SNAPs amplitude (*P* = 0.029), longer SNAPs latency (*P* = 0.0013), and lower SCV (*P* = 0.00095) than those of the normal mice. These findings indicated that the mice with SNI had the most severe sensory disturbance with allodynia including demyelination of the sciatic nerve. The DN (db/db) model showed significantly longer SNAPs latency (*P* = 0.023) and lower SCV (*P* = 0.00063) than those of the normal mice, indicating that this model had moderate sensory disturbance with allodynia. In contrast, SNAPs amplitude, SNAPs latency, and SCV were not significantly different in the CIPN (paclitaxel) model even though the mice had allodynia as evidenced by the plantar and von Frey test results shown in Fig. 4. These results indicated that the CIPN (paclitaxel) mouse model showed slight sensory disturbance electrophysiologically but had allodynia.

#### 3.1.2 Compound muscle action potentials

**Fig. 3:**
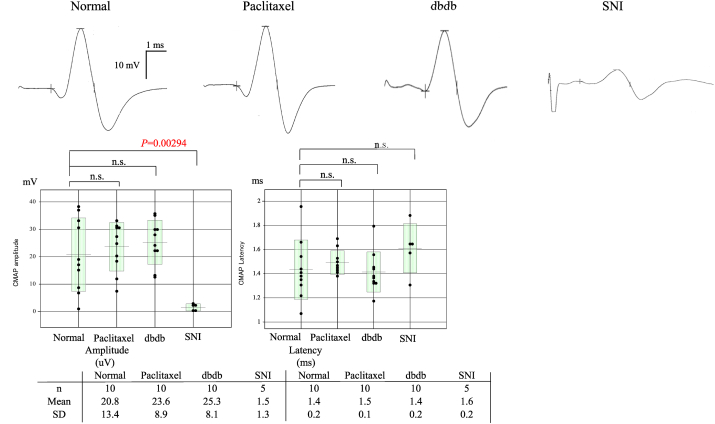
CMAPs in the sciatic nerve. Latency and amplitude of CMAPs of normal and NP mice models of CIPN (paclitaxel), DN (db/db), and EN (SNI) are shown. Only the EN (SNI) model had significantly lower motor-evoked potential amplitude (*P* = 0.00294) than that of normal mice. The data of the CMAPs parameter were analysed with one-way ANOVA. Multiple comparisons were performed with Dunnett's multiple comparison, as appropriate, following the Kruskal-Wallis test. CIPN, chemotherapy-induced peripheral neuropathy; CMAPs, compound motor action potentials; DN, diabetic neuropathy; EN, entrapment neuropathy; NP, neuropathic pain; n.s., not significant; SNI, spared nerve injury.

We measured CMAPs in the sciatic nerve (Fig. 1a, b, d). CMAPs latency and amplitude in the normal and NP mice models of CIPN (paclitaxel), DN (db/db), and EN (SNI) are shown in Fig. 3.

**Fig. 4:**
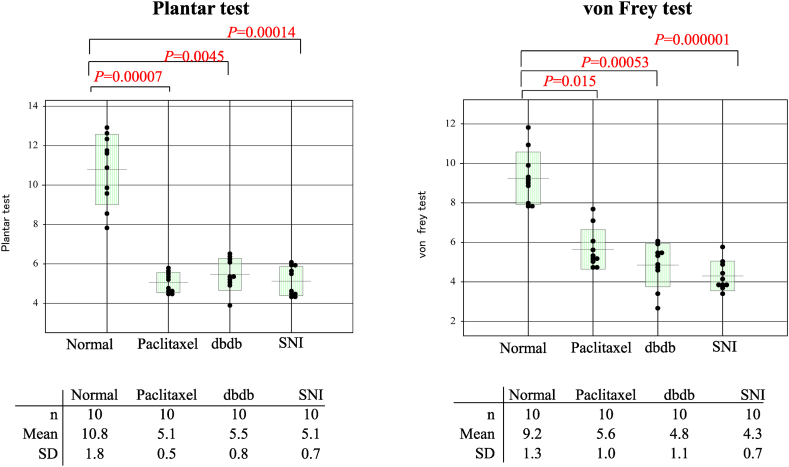
Assessment of tactile and thermal sensitivity as determined by plantar withdrawal test and von Frey withdrawal test. All three neuropathic pain models showed significantly lower thresholds than normal mice in the plantar test and von Frey test, indicating that these three models had allodynia and neuropathic pain. The data of the PWL and PWT parameters were analysed with one-way ANOVA. Multiple comparisons were performed with Dunnett's multiple comparison, as appropriate, following the Kruskal-Wallis test. PWL, paw withdrawal latency; PWT, paw withdrawal threshold; SNI, spared nerve injury.

Only the EN (SNI) model had significantly lower motor-evoked potential amplitude (*P* = 0.00294) than that of the normal mice, indicating that the NP mice models had sensory disturbance with allodynia including demyelination of the sciatic nerve. However, the other two NP models, the CIPN (paclitaxel) and DN (db/db) models, only showed sensory disturbance but no loss of motor function.

### 3.2 Plantar test/von Frey test

We conducted plantar withdrawal and von Frey withdrawal tests to confirm the presence of thermal hyperalgesia and mechanical allodynia of each NP model. All three NP models had significantly lower thresholds in the plantar test and von Frey test, indicating that all three models had allodynia and NP (Fig. 4).

## 4 Discussion

There are no ways to directly measure pain in animals, no matter the species. Historically, pain assessment in animals has mainly been based on the reflex withdrawal response to a sensory stimulus rather than by directly measuring more complex, directly relevant animal behaviour that might infer the presence of spontaneous pain [12]. Regardless, evaluating ‘pain’ itself, which includes unpleasant sensory and emotional experiences, remains difficult when only pain withdrawal, reaction, and behaviour can be observed in animal models. Contrastingly, electrophysiological assessment of sensory and motor nerves is objective, and the amount peripheral nerve damage can be quantified in NP animal models and is not influenced by complex unpleasant sensory and emotional sensations. However, the detailed methodology, stimulation intensity, measurement and stimulation points, and normative values have varied widely in each previous report and in each animal model [[3], [4], [5], [6], [7],^10^,^11^,[14], [15], [16], [17], [18], [19], [20],^22^,^23^,^25^,^28^]. In the present study, we presented an established method of electrophysiological analysis of SNAPs, SCV, and CMAPs in the sciatic nerve and showed the electrophysiological characteristics and normative values of experimental NP mice models of DN, CIPN, and EN, which are the most frequently and conventionally used experimental models of NP [5,21,24,27].

Previously published articles reporting the methodology and outcome of SNAPs and SCV evaluation in mice and rats have shown the assessed sensory nerves and area, stimulus intensities and frequencies, and normative values [2,7,10,14,15,[17], [18], [19], [20],^22^,^23^,^25^,^28^]. Several articles reported that nerve conduction in the dorsal caudal sensory nerve could be recorded in the tail using several stimulation protocols and different types of recording needle electrodes [2,7,15,17,18,20,22,25,28]. Other papers reported that the sural sensory nerve could be stimulated with small electrodes placed under the external malleolus on the lateral side of the ankle [2]. Sciatic nerve recording for SNAPs and NCV analyses was also reported in which the stimulating points were the sciatic nerve directly and the ankle segment and bottom of the foot, and in which the recording points were the sciatic notch and sciatic nerve directly [2,10,15,19,22,23]. The reference intervals of SNAPs differed in each article, and some papers did not state the stimulus intensity and frequency. The reference intervals of SCV were also different in each article because of the differences in methodologies (Table 1) [2,7,14,15,[17], [18], [19], [20],^22^,^23^,^25^,^28^].

**Table 1:** Electrophysiological studies of peripheral nerve damage in db/db, PI-NP, SNI, and other models.

1st Author [reference]	Year	Experimental model	SNAPs: examined nerve and average amplitude/velocity	CMAPs: examined nerve/muscle and average amplitude/velocity	SCV: examined nerve and average velocity	Other data
Kan HW [2]	2018	Transthyretin knock-in mice	**Sural nerve**	**Sciatic nerve/plantar m.**	–	–
			AVG amp: 636 ± 198–966 ± 344 μV	AVG amp: 4.00 ± 1.52–8.01 ± 2.25 mV		
Liu Y [7]	2017	24-week-old db/db mice	**Tail nerves**	**Tibial nerve/intrinsic foot m.**	**Tail nerves**	–
			AVG amp: around 32 μV	AVG amp: around 1.3 mV	AVG vel: 21.6 ± 0.36 m/s	
Hamad MN [10]	2022	SD rat Nerve crush model	–	**Sciatic nerve/gastrocnemius** AVG amp: 4.1 ± 3.4 mV	–	Hook type electrodes
						**Normal rat CMAPs**
						AVG amp: 25.0 ± 9.7 mV
Tarhzaoui K [14]	2008	Diabetes Wistar rats (3 months old)	**External saphenous nerve** AVG amp: around 17 μV AVG vel: around 2.6 ms	**Distal tibial posterior nerve/first interosseous** AVG amp: around 12.8 mV AVG vel: around 39.5 m/s	**External saphenous nerve** AVG vel: around 51.5 m/s	**Normal rat CMAPs**
						AVG amp: around 14.5 mV
						**Normal rat SNAPs**
						AVG amp: around 16 μV
Walsh ME [15]	2015	Aging mice (4–32 months old)	**Tail nerve**	**Tail nerve**	**Tail nerve** AVG vel: around 24–34 m/s	–
			AVG amp: around 65–116 μV	AVG amp: around 2.2–3.2 mV		
			**Sural nerve**	**Sciatic nerve**		
			AVG amp: around 130–185 μV	AVG amp: around 7.5–16.5 mV		
Caillaud M [17]	2022	PI-NP mice	**Caudal nerve**	–	**Caudal nerve**	Needle electrodes
			AVG amp: around 31–47 mV		AVG vel: around 24 m/s	
Nodera H [18]	2012	Mice model exposed to ranolazine	**Tail nerves** Exact number not listed	**-**	-	–
Homs J [19]	2011	Non-obese diabetic mice (47 days after induction)	**Digital nerve** AVG amp: 32.6 ± 2.6 μV AVG vel: 43.3 ± 1.6 m/s	**Sciatic nerve/anterior tibialis m.**	-	Microneedle electrodes
				AVG amp: 52.4 ± 2.4 mV		
				**Sciatic nerve/plantar m.**		
				AVG amp: 6.7 ± 0.5 mV		
Leandri M [20]	2012	PI-NP mice	–	–	**Tail nerves**	–
					21.3 ± 1.81–25.69 ± 2.29 m/s	
Sullivan KA [22]	2007	db/db mice	–	–	**Tail nerve**	**Sciatic-tibial nerve MCV**
					AVG vel: 22.1 ± 0.4 m/s	AVG vel: 15.6 ± 0.9 m/s
Wang L [23]	2011	16-week-old db/db mice	–	–	**Sciatic nerve**	**Sciatic nerve/intrinsic foot m. MCV**
					AVG vel: around 30–33 m/s	AVG vel: around 28–31 m/s
Boehmerle W [25]	2014	PI-NP mice	**Caudal nerve**	**-**	**Caudal nerve**	**-**
			AVG amp: around 16–32 μV		Exact number not listed	
Cavaletti G [28]	2024	PI-NP mice	**Caudal nerve**	–	**Caudal nerve**	–
			AVG amp: around 20–120 μV		AVG vel: around 36–52 m/s	
			**Digital nerve**		**Digital nerve**	
			AVG amp: around 30–70 μV		AVG vel: around 23–25 m/s	

db/db, C57BLKS/J lar- + Leprdb/+Leprdb (db/db) mice; PI-NP, paclitaxel-induced neuropathic pain; SNI, spared nerve injury; SNAPs, sensory nerve action potentials; CMAPs, compound motor action potentials; SCV, sensory conduction velocity; MCV, motor conduction velocity; AVG amp, average amplitude, AVG vel, average velocity; m., muscle.

The assessment of CMAPs in the previous reports had similar issues. The most assessed motor nerve was the sciatic nerve in which recording was from the plantar/sural muscles and stimulation was applied at the sciatic notch. However, stimulus intensity and frequency were also different or not described in the papers, and they varied with the type of electrodes used, as did the outcome in each report [2,7,10,14,15,19]. Many similar articles were published on electrophysiological examinations of NP models. We show the variability of the data, species, and analysed nerves from these articles in Table 1. However, we could not verify either the detailed methodology or certain issues in each article because information on the methodology used was not adequately described [2,10,15,19]. Therefore, we focused on the detailed methodology in the present study for the first time, to our knowledge.

We presented a reproducible method of electrophysiological analysis of SNAPs, SCV, and CMAPs to obtain stable values in the analysis of sciatic nerve function. From our preliminary study, stimulation and recording at the proximal point of the sciatic nerve using needle electrodes had also stimulated muscles near the sciatic nerve, and thus, the SNAPs, SCV, and CMAPs data had included several artefacts reflected on the electromyogram. In addition, an unstable length between the positive and negative electrodes during the examination caused statistical variability in each mouse group. Therefore, in the present study, we used hooked-type electrodes that fixed the width of the±electrodes at the proximal sciatic nerve for stimulating and recording, and we could also raise the sciatic nerve slightly from the surrounding muscles to avoid muscle artefacts during recording and stimulation. When we used these specific electrodes and stimulating and recording points as described in detail in the Methods section, the data no longer varied statistically in each group.

We used the three most common different mouse models of NP and showed the characteristics of peripheral nerve dysfunction in the NCS [5,21,24,27]. C57BLKS/J lar- + Leprdb/+Leprdb (db/db) mice, which are a diabetes model, have the characteristics of loss of sensation and abnormal sensation such as numbness [[21], [22], [23], [24]]. CIPN mice have the characteristics of axonal degeneration, decrease of NCV, and abnormality of pain sensitivity [17,20,24,25,27,28]. In addition, the SNI model has the characteristics of mechanical allodynia, upregulation against heat stimulation, and chronic pain [10,12]. In the clinical setting, most of the patients with DN have the symptom of loss of sensation in the distal portion of their extremities [21], whereas most with CIPN complain of numbness in the hands and feet [27], and patients with sciatica have the symptoms of severe lower limb radiculopathy and numbness [33]. The pathologies are different in each model even if they are uniformly defined as peripheral neuropathy.

This is the first report to reveal the electrophysiological differences in each NP model. While we know of no articles showing the electrophysiological differences in DN, CIPN, and sciatica in the clinical setting, we think that the SNAPs, CMAPs, and SCV in these experimental models have the possibility to reflect DN, peripheral neuropathy following anticancer drug therapy, and sciatica caused by lumbar spinal stenosis or lumbar herniation in patients.

A previous article reported an electrophysiological study of peripheral nerve damage in C57BLKS/J lar- + Leprdb/+Leprdb (db/db) mice in a diabetes model [15,22,23]. SNAPs in tail nerves were significantly reduced in 24-week-old db/db mice (average: 33 μV) compared with those in control mice (average: 54 μV) [15,22,23]. SCV of these 24-week-old db/db mice also decreased significantly (21.6 ± 0.36 m/s) compared to that of the control mice. Although CMAPs also decreased significantly in these 24-week-old mice (average amplitude: db/db; 1.3 mV, control: 3.0 mV), they did not decrease in the 6-week-old db/db mice [7,15,22,23]. The db/db mice in our model were 11 weeks old, and thus, the sciatic nerve showed more mild degeneration in the electrophysiological analysis.

Some articles reported that SNAPs, SCV, and CMAPs in a PI-NP model showed the effects of CIPN. A length-dependent axonal sensory neuropathy caused by paclitaxel administration can induce peripheral neuropathy [17,20,25,28]. SNAPs in PI-NP mice were 33–36 μV and were significantly decreased compared to those of 45–50 μV in the normal mice [17,20,25,28]. Other papers also reported that SNAPs amplitude in the caudal nerve decreased significantly in PI-NP mice [17,25]./

Severe peripheral nerve damage has been reported electrophysiologically in the SNI surgery model, which represents EN [5,10,12,28]. SNI is also a model of partial denervation, whereby injury to the common peroneal and tibial nerves produces a consistent, reproducible, and tactile hypersensitivity in the skin innervated by the spared, intact sural nerve [5,10]. In the present study, although the difference in SCV between the paclitaxel group and normal group was not significant, it was significantly lower in the db/db group and SNI group, suggesting that the SNI group had a high degree of conduction block due to local ischemia, whereas the db/db group had severe axonal degeneration of the large-diameter fibres of the sensory nerve. The involved lesions in the paclitaxel group were at most probably limited to small-diameter fibres and were not reflected in the SCV [2,7,10,14,15,[17], [18], [19], [20],^22^,^23^,^25^,^28^].

However, electrophysiological assessment of the sciatic nerve has not been reported with clear data and detailed methodologies. We revealed that the amplitudes of CMAPs and SNAPs and SCV were significantly decreased compared to those seen in normal mice. Compared to other peripheral neuropathy models, this was most severe in the peripheral neuropathy mice model (Fig. 2, Fig. 3). Although electrophysiologic study is useful in quantitatively assessing the degree of nerve fibre damage, the degree of allodynia present makes full assessment difficult. However, it has been established that damage limited to small-diameter fibres may be difficult to assess even in SNAPs, which are more sensitive than CMAPs. Our findings reflect the overall state of nerve dysfunction, and we hypothesize that smaller pain-conducting fibres are likely to be affected to an equal or greater extent than the larger fibres assessed here. These distinctions align with clinical observations in DN and CIPN, in which motor function is typically preserved.

## 5 Conclusions

The present study showed our established methods of electrophysiological analysis of CMAPs, SNAPs, NCV, and SCV in the sciatic nerve of various mice models. We also revealed the electrophysiological characteristics of experimental NP mice models of DN, CIPN, and EN.

## CRediT authorship contribution statement

**Hidenori Suzuki:** Writing – review & editing, Writing – original draft, Funding acquisition, Formal analysis. **Masahiro Funaba:** Writing – original draft, Methodology. **Sayaka Ogi:** Methodology, Investigation, Formal analysis, Data curation, Conceptualization. **Kosei Matsumoto:** Resources, Formal analysis, Data curation, Conceptualization. **Norihiro Nishida:** Writing – review & editing, Writing – original draft. **Kazuhiro Fujimoto:** Writing – original draft. **Takashi Sakai:** Writing – review & editing, Writing – original draft. **Kenji Nishikawa:** Writing – review & editing, Writing – original draft, Project administration, Methodology, Investigation, Formal analysis, Data curation, Conceptualization.

## Data availability statement

The authors confirm that the data supporting the findings of this study are available within the article.

## Ethics approval and consent to participate

The Animal Care and Use Committee of Pharmaceuticals Research Laboratory, UBE Corporation, approved all animal care and experimental procedures (approval numbers: 22135, 22141–22143). All methods were reported in accordance with ARRIVE guidelines (https://arriveguidelines.org), and all methods were carried out in accordance with relevant guidelines and regulations.

## Consent for publication

Not applicable.

## Funding

This work was supported by 10.13039/501100003478MHLW FG Program Grant Number JPMH22FG2001 to Hidenori Suzuki.

## Declaration of competing interest

The authors declare that they have no known competing financial interests or personal relationships that could have appeared to influence the work reported in this paper.
